# Liver segmental volume and attenuation ratio (LSVAR) on portal venous CT scans improves the detection of clinically significant liver fibrosis compared to liver segmental volume ratio (LSVR)

**DOI:** 10.1007/s00261-020-02834-7

**Published:** 2020-11-06

**Authors:** V. C. Obmann, C. Marx, J. Hrycyk, A. Berzigotti, L. Ebner, N. Mertineit, Ch. Gräni, J. T. Heverhagen, A. Christe, A. T. Huber

**Affiliations:** 1 Department of Diagnostic, Interventional and Pediatric Radiology, Inselspital, Bern University Hospital, University of Bern, Freiburgstrasse 10, 3010 Bern, Switzerland; 2 Hepatology, Department of Visceral Surgery and Medicine, Inselspital, Bern University Hospital, University of Bern, Bern, Switzerland; 3 Department of Cardiology, Inselspital, Bern University Hospital, University of Bern, Bern, Switzerland

**Keywords:** Computed tomography, Liver cirrhosis, Fibrosis, Computer-assisted image processing, Computer-assisted diagnosis

## Abstract

**Background:**

The aim of this proof-of-concept study was to show that the liver segmental volume and attenuation ratio (LSVAR) improves the detection of significant liver fibrosis on portal venous CT scans by adding the liver vein to cava attenuation (LVCA) to the liver segmental volume ratio (LSVR).

**Material and methods:**

Patients who underwent portal venous phase abdominal CT scans and MR elastography (reference standard) within 3 months between 02/2016 and 05/2017 were included. The LSVAR was calculated on portal venous CT scans as LSVR*LVCA, while the LSVR represented the volume ratio between Couinaud segments I-III and IV-VIII, and the LVCA represented the density of the liver veins compared to the density in the vena cava. The LSVAR and LSVR were compared between patients with and without significantly elevated liver stiffness (based on a cutoff value of 3.5 kPa) using the Mann–Whitney U test and ROC curve analysis.

**Results:**

The LSVR and LSVAR allowed significant differentiation between patients with (*n* = 19) and without (*n* = 122) significantly elevated liver stiffness (*p* < 0.001). However, the LSVAR showed a higher area under the curve (AUC = 0.96) than the LSVR (AUC = 0.74). The optimal cutoff value was 0.34 for the LSVR, which detected clinically increased liver stiffness with a sensitivity of 53% and a specificity of 88%. With a cutoff value of 0.67 for the LSVAR, the sensitivity increased to 95% while maintaining a specificity of 89%.

**Conclusion:**

The LSVAR improves the detection of significant liver fibrosis on portal venous CT scans compared to the LSVR.

## Introduction

Early detection of clinically significant liver fibrosis is of utmost importance in preventing disease progression and the development of hepatocellular carcinoma (HCC) [1]. In addition to leading to improved patient prognosis, early prevention and treatment might lower healthcare costs [2]. Ultrasound and magnetic resonance (MR) elastography allow noninvasive grading of significant liver fibrosis in patients with suspected chronic liver disease [3], but patients often present with already advanced liver fibrosis and cirrhosis. Many CT scans in radiology departments are performed for a multitude of reasons unrelated to chronic liver disease (CLD). Good and reliable quantitative liver fibrosis scores obtainable from routine portal venous CT scans are therefore highly desirable.

Most CT methods for detecting liver fibrosis are qualitative and therefore reader-dependent techniques [4]. Several quantitative methods allow for the detection of advanced fibrosis on CT scans, such as the caudate–right lobe ratio (CRL-R) and liver vein diameters [5, 6], CT texture analysis [7, 8], liver surface nodularity [9, 10], splenic volume and liver segmental volume ratio (LSVR) [11, 12]. Recently, improved predictions of significant liver fibrosis were achieved on abdominal CT scans with the new liver imaging morphology and attenuation fibrosis score (LIMA-FS), which is a combination of the CRL-R with a liver vein to cava attenuation (LVCA) score [13].

Since the LVCA is simple and easy to calculate, we hypothesized that the LVCA might also increase the performance of the LSVR in detecting liver fibrosis. The aim of this proof-of concept study was to show that the detection of significant liver fibrosis on abdominal CT scans may be improved with the use of the liver segmental volume and attenuation ratio (LSVAR), which is as a combination of the LVCA and LSVR.

## Materials and methods

### Study population

This prospective cross-sectional study was approved by the institutional review board (Kantonale Ethikkommission Bern, IRB number 282-15) and conducted after obtaining written informed consent from the patients.

The inclusion criteria were age between 18 and 70 years as well as a portal venous phase abdominal CT scan and gradient echo-based MR elastography exam (reference standard) performed within 3 months. The indications for CT scans were trauma (*n* = 22), search for inflammation (*n* = 34), ileus (*n* = 13), nonliver tumor (*n* = 43), HCC screening (*n* = 15) and abdominal pain (*n* = 14). The exclusion criteria included solid liver lesions > 2 cm, portal vein thrombosis, prior liver surgery and transplantation. After applying the inclusion and exclusion criteria, 148 consecutive patients were included from 02/2016 to 05/2017.

**Fig. 1:**
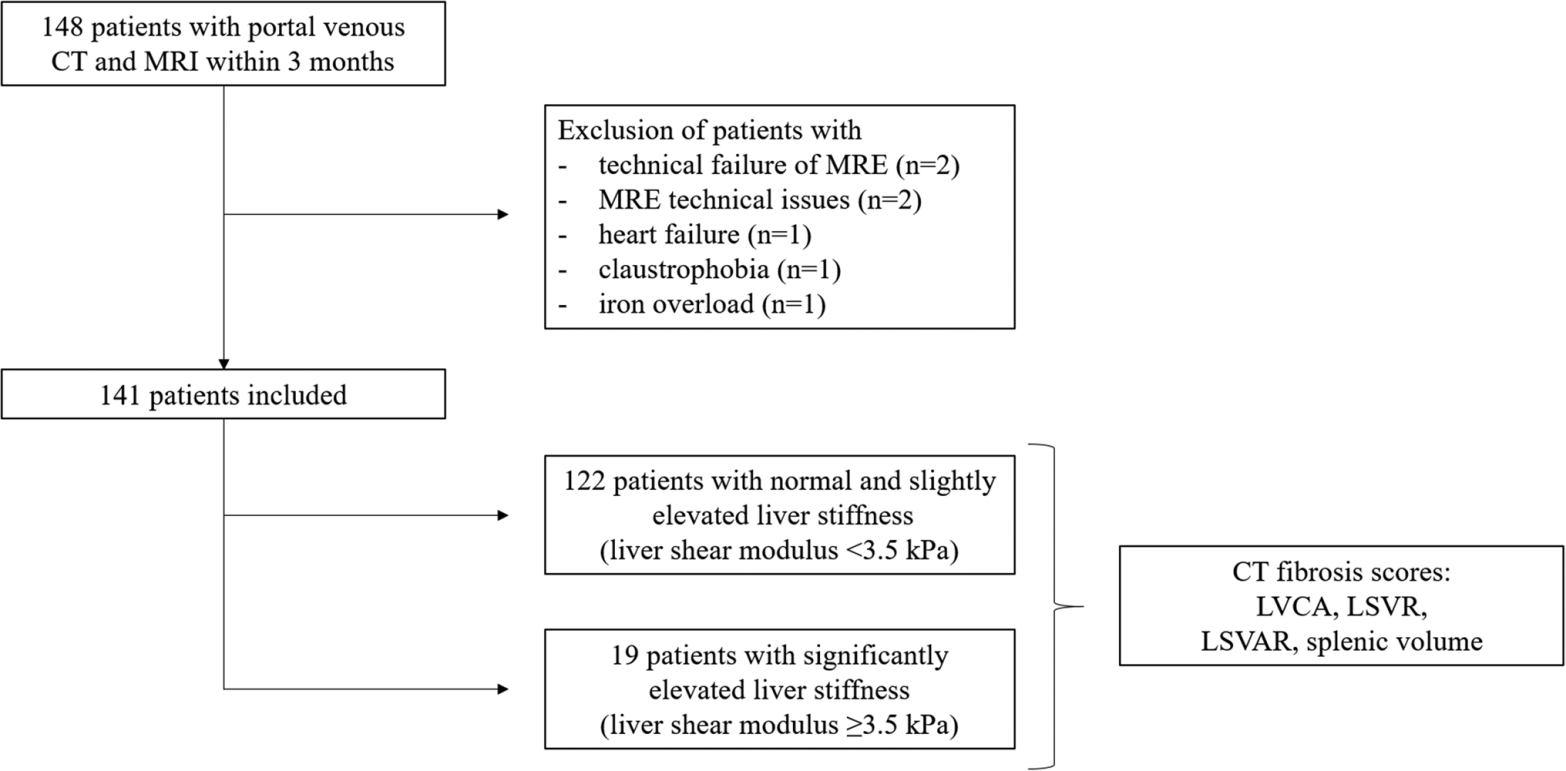
Patient flowchart. A total of 152 patients with portal venous abdominal CT scans were included in the study. MRI scans from six patients were excluded. MRI scans from 146 patients were included in the analysis and separated based on their liver stiffness (< 3.5 kPa, *n* = 122 and ≥ 3.5 kPa, *n* = 24). All CT fibrosis scores as well as splenic volumes were assessed for all patients

Seven patients were excluded because of technical failure during MR elastography (*n* = 6) or early cessation of the MR exam due to claustrophobia (*n* = 1), resulting in a total study population of 141. A total of 122 patients had a liver stiffness < 3.5 kPa, while 19 patients had a stiffness of 3.5 kPa, consistent with clinically significant liver fibrosis (corresponding to a fibrosis stage ≥ F2) (Fig. 1). Clinical information (BMI, comorbidities, smoking and alcohol habits) and laboratory test results (liver enzymes, coagulation results and APRI) were recorded. Data from this patient population have already been published in other research studies [13–15].

### CT imaging and postprocessing

CT scans were acquired on Siemens Somatom Definition Flash, Definition Edge (Siemens Healthineers, Erlangen, Germany) and Philips Brilliance 64 (Philips, Best, Netherlands) scanners with a pitch of 0.8 and a detector collimation of 0.6. The acquisitions were performed with attenuation-based kV selection and automated mAs adaption using references of 100 kVp and 150 mAs. Portal venous phase acquisitions were performed 75 s after the injection of 110 ml of contrast agent (Ultravist® 370, Bayer AG, Leverkusen, Germany, Xenetix® 300, Guerbet, Villepinte, France, Iomeron® 300, Bracco, Milan, Italy, flow rate 2 ml/s). One-millimeter axial slices were reconstructed with an increment of 1 mm in a liver parenchyma window using a vendor-specific iterative reconstruction algorithm (Saphire level 3 and iDose level 3).

**Fig. 2:**
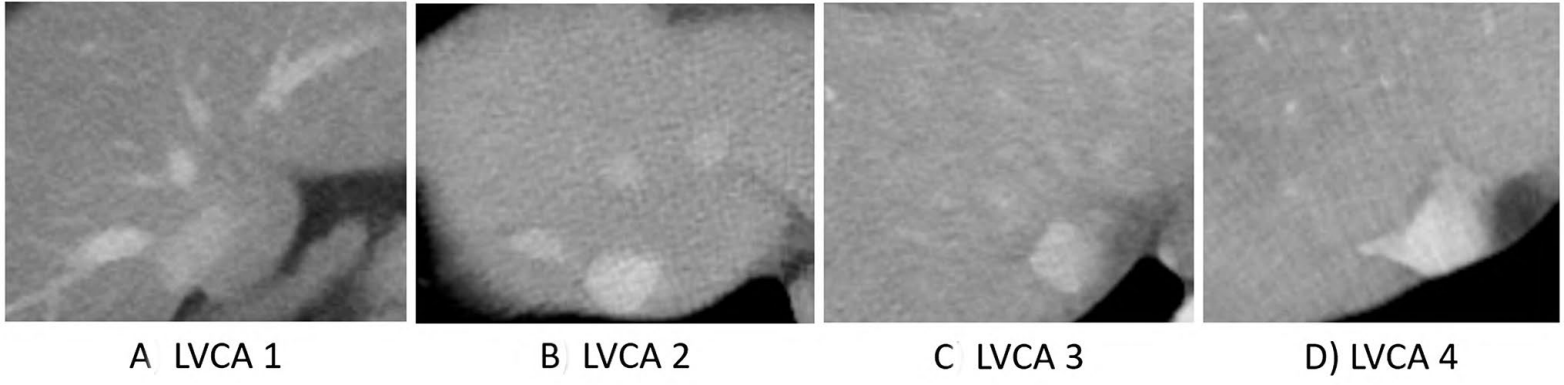
Liver vein to cava attenuation (LVCA) classification. **a** Category 1: liver veins are hyperattenuated compared to the inferior vena cava (IVC), **b** 2: liver veins are isoattenuated, **c** 3: liver veins are hypoattenuated, and **d** 4: liver veins have no visible contrast. *LVCA* liver vein to cava attenuation, *IVC* inferior vena cava

CT images were evaluated by a radiologist (A.T.H.) with 7 years of experience on a dedicated reading workstation (MDCC-6230, Barco, Kortrijk, Belgium) using the Picture Archiving and Communication System (PACS) (IDS7, SECTRA, Linköping, Sweden). The LVCA was determined by comparing the attenuation of the liver veins to the inferior vena cava (IVC) as described previously [13]. The LVCA is an ordinal score based on the density of the liver veins 1 cm proximal to the liver vein confluence compared with the vena cava 1 cm below the liver vein confluence. Assessment is based on a visual comparison of the liver veins and the inferior vena cava. In the case of similar attenuation, a region of interest is drawn in the liver veins, 1 cm proximal to the liver vein confluence and in the inferior vena cava 1 cm below the liver vein confluence. If the mean density of the three liver veins is within ± 20 HU of the density of the vena cava, the liver veins are defined as isoattenuating to the vena cava (LVCA score 2). Otherwise, the liver veins are called hyperattenuating (LVCA score 1) or hypoattenuating (LVCA score 3). If the liver veins are not contrasted at all, this results in an LVCA score of 4 (Fig. 2).

Liver volumetry was performed using the liver analysis application in Philips Intellispace Portal (Philips, Best, Netherlands) volumetry software by a second radiologist (C.M.) with 2 years of experience in liver imaging. Liver contours were recognized by the software and manually corrected if needed. In a next step anatomical landmarks (inferior vena cava, right and left portal bifurcation, right and mid hepatic vein, umbilical fissure, tip of the left liver, superficial and deep ligamentum venosum) were defined based on the landmarks the software calculated the volume of each liver segment as well as the sum for volume for the left (segments I-III) and right (segments IV to VIII) liver lobes. The LSVR then was calculated as described in the literature [12]:

$$LSVR = \frac{{Volume\;segments\;I - III}}{{Volume\;segments\;IV - VIII}}$$

**Fig. 3:**
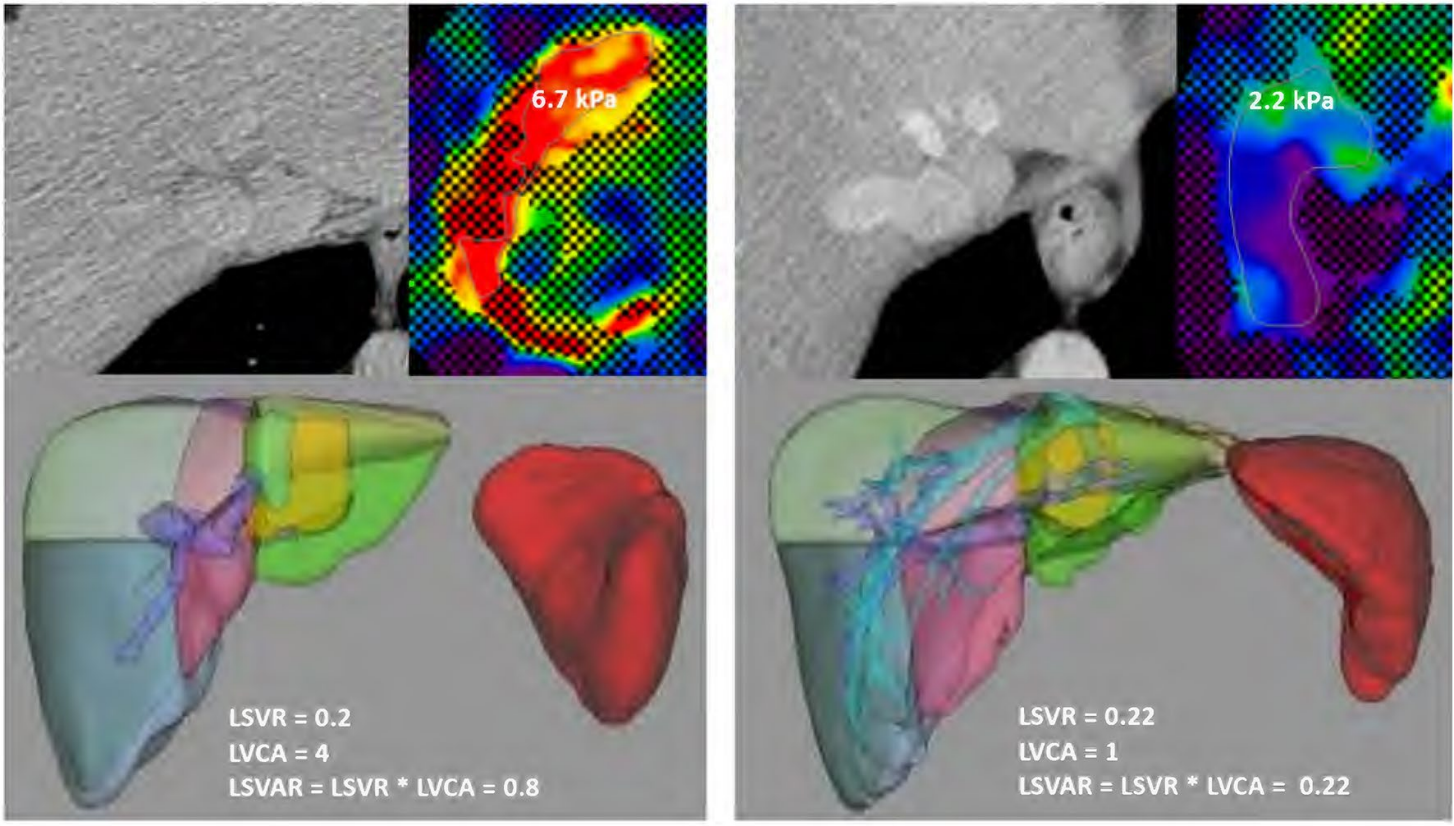
Patient examples with and without clinically significant increased liver stiffness demonstrating the LSVR and LSVAR. On the left side, CT images from a cirrhotic patient (liver stiffness of 6.7 kPa) with hypertrophic left and caudate lobes as well as noncontrasted liver veins (LVCA 4) are shown, resulting in an LSVR = 0.2 and LSVAR = 0.8. On the right side, CT images from a patient with normal liver stiffness are shown (2.2 kPa). All liver fibrosis measures were within normal limits; LSVR = 0.22, LSVAR = 0.22 and LVCA = 1. *LVCA* liver vein to cava attenuation, *LSVR* liver segmental volume ratio, *LSVAR* liver segmental volume and attenuation ratio

The LSVAR was then calculated as LSVR * LVCA (Fig. 3). The total time to determine the LSVAR was approximately ten minutes per patient, depending on the manual adjustments for liver organ margins.

To compare the performance of semiautomated and manual segmentation, segments I to III and segments IV to VIII were segmented manually in a second analysis using the region growing tool in all patients with a liver stiffness > 2.8 kPa.

### MR imaging technique and imaging analysis

The patients were examined with a 3-T MR system (Verio, Siemens Healthineers, Erlangen, Germany) in a fasting state (> 6 h). A pneumatic driver (Resoundant, Rochester, MN, USA) was placed on the right upper quadrant to transmit shear waves by continuous acoustic vibrations at 60 Hz. The liver shear modulus in kPa in the right upper liver lobe was determined with a gradient echo-based elastography sequence (WIP package 622 provided by Siemens Healthineers, 3 single-slice acquisitions with 5  mm slice thicknesses) using the 95% confidence map of stiffness. The MR elastography images were read by a radiologist who was blinded to the results of the CT scans. MR elastography-based stiffness assessments were used as a reference standard for liver fibrosis graduation. A cutoff value of ≥ 3.5 kPa for clinically significant liver fibrosis was used, corresponding to a Metavir stage ≥ F2 in histology and a cutoff of 8 kPa in transient elastography (FibroScan®) [16].

### Statistical analysis

Analysis was performed with the statistical software package R version 3.4.1 [17] and GraphPad Prism (Version 7.1, GraphPad Software Inc, CA, USA). ROC curve analysis was used to compare the performances of CT fibrosis scores to predict significantly elevated liver stiffness. Areas under the curves (AUCs) and 95% confidence intervals were calculated. Cutoff values were chosen based on Youden’s index. Clinical parameters were compared between patient groups using the Mann–Whitney U test for continuous variables and Fisher’s exact test for categorical variables. To determine the interrater reliability of volumetry measurements, another radiologist (J.H., 3 years of experience) measured segmental volumes in 20 randomly selected patients, and one-way consistency intraclass correlation coefficients (ICCs) were then calculated for the LVCA, LSVR and LSVAR. To compare the performance of semiautomated and manual segmentation, ROC curve analysis was repeated using the LSVR and LSVAR based on the manual segmentation. For ICC results, classifications from 0.4 to 0.59 are considered as fair, those from 0.6 to 0.74 as good, and those from 0.75 to 1.00 are considered excellent [18].

## Results

### Patient characteristics

Based on MRE there were 90 patients with a normal liver stiffness (< 2.5 kPa), 20 patients with liver stiffness ≥ 2.5–2.9 kPa (normal or chronic inflammation), 12 patients with liver stiffness ≥ 2.9–3.5 kPa (F1-2), 5 patients with liver stiffness ≥ 3.5–4 kPa (F2-3), 4 patients with liver stiffness ≥ 4–5 kPa (F3-4) and 10 patients with liver stiffness ≥ 5 kPa (F4). The patient characteristics are shown in Table 1. Patients with elevated liver stiffness ≥ 3.5 kPa had significantly higher levels of liver enzymes (AST 37.0 [31.5–68.0] vs. 22.0 [19.3–25.8], *p* < 0.001, ALT 30.0 [25.0–46.0] vs. 23.0 [17.0–35.8], *p* = 0.015; GGT 109.0 [70.0–150.0] vs. 23.0 [17.0–38.0], *p* < 0.001) as well as bilirubin (19 [13.5–35.0] vs. 7.0 [5.0–12.0], *p* < 0.001) and APRI (0.9 [0.6–29] vs. 0.2 [0–0.3], *p* = 0.033) but lower Quick values (71.5 [63.2–90.5] vs. 100.0 [99.0–100.0], *p* < 0.001). Two patients had ascites. There were more smokers (58% vs. 16%, *p* < 0.001) and patients who consumed alcohol daily (72% vs. 6%, *p* < 0.001) among the patients with elevated liver stiffness.

**Table 1:** Patient characteristics

	Normal to slightly increased liver stiffness (shear modulus < 3.5 kPa)	*n*	Significantly increased liver stiffness (shear modulus ≥ 3.5 kPa)	*n*	*p*–value
Age, years	53.4 [42.9–62.3]	122	55.9 [54.0–66.1]	19	0.134
Male, %	62 (51%)	122	16 (84%)	19	< 0.001**
Shear modulus, kPa	214 [187–254]	122	481 [378–574]	16	< 0.001**
Tobacco use, *n* (%)	19 (16%)	121	11 (58%)	19	< 0.001**
Alcohol consumption, *n* (%)	8 (6%)	121	13 (72%)	18	< 0.001**
Diabetes, *n* (%)	8 (7%)	120	7 (37%)	19	0.001*
Hypertension, *n* (%)	26 (22%)	121	9 (47%)	19	0.023*
BMI, kg/m^2^	25.3 [22.9–29.0]	122	27.7 [25.0–33.4]	19	0.072
PDFF, %	7% [5–11%]	121	9 [6–17]	18	0.710
AST, U/l	22.0 [19.3–25.8]	54	37.0 [31.5–68.0]	15	< 0.001**
ALT, U/l	23.0 [17.0–35.8]	70	30.0 [25.0–46.0]	15	0.015*
GGT, U/l	23.0 [17.0–38.0]	61	109.0 [70.0–150.0]	16	< 0.001**
Alkaline phosphatase, U/l	72.0 [54.0–83.5]	55	97.0 [64.5–118.5]	15	0.041*
Bilirubin, μmol/l	7.0 [5.0–12.0]	47	19 [13.5–35.0]	15	< 0.001**
Albumin	36 [34.8–38.0]	40	35 [30.0–38.0]	16	0.405
Dyslipidemia	13 (11%)	121	3 (16%)	19	0.457
Quick, %	100.0 [99.0–100.0]	59	71.5 [63.2–90.5]	16	< 0.001**
APRI	0.2 [0–0.3]	30	0.9 [0.6–29]	9	0.033*
Creatinine, μmol/l	76.0 [67.0–91.0]	89	71.0 [65.0–82.0]	18	0.326
Chronic renal insufficiency	1 (1%)	120	1 (5%)	19	0.256
≥ 1 medication(s) per day	29 (24%)	121	15 (79%)	19	< 0.001**

Values are reported as the median and interquartile range or the absolute number (n) with the percentage of study group. *P* values were calculated using the Mann–Whitney U test or Fisher’s exact test, as appropriate. Comparisons between the two patient groups are indicated with * if *p* < 0.05 and ** if *p* < 0.001

*BMI* body mass index, *PDFF* proton density fat fraction, *AST* aspartate aminotransferase, *ALT* alanine aminotransferase, *GGT* gamma-glutamyltransferase, *APRI* aspartate aminotransferase-to-platelet ratio index

Of the 19 patients with elevated liver stiffness (shear modulus ≥ 3.5 kPa), 18 had known liver fibrosis (3 patients with a histology fibrosis grade F2, 6 patients with a grade of F3 and 9 patients with a grade of F4 on histology or clinically established diagnosis of liver cirrhosis), while chronic liver disease was not known in 1 patient who was lost to follow-up (cancer patient without liver metastasis). The etiology of liver disease in those patients was as follows: viral hepatitis (*n* = 9), alcohol induced liver disease (*n* = 2), NAFLD/NASH (*n* = 3), cryptogenic liver cirrhosis (F4) on histology (*n* = 1) and unknown in the one patient who was lost to follow-up. The median time interval between biopsy and MRE was 18 months [IQR 3.5–26.5 months]. The median MRE ROI size was 3184 mm^2^ [IQR 2129–4643 mm^2^].

Of the 122 patients without elevated liver stiffness (shear modulus < 3.5 kPa), 2 had liver biopsy with a diagnosis of NAFLD/NASH (1 patient with a histology fibrosis grade of F1 and one patient with a grade of F2), 5 patients had chronic viral hepatitis without known liver fibrosis, and the remaining 115 patients had no known chronic liver disease.

### Liver fibrosis detection

**Fig. 4:**
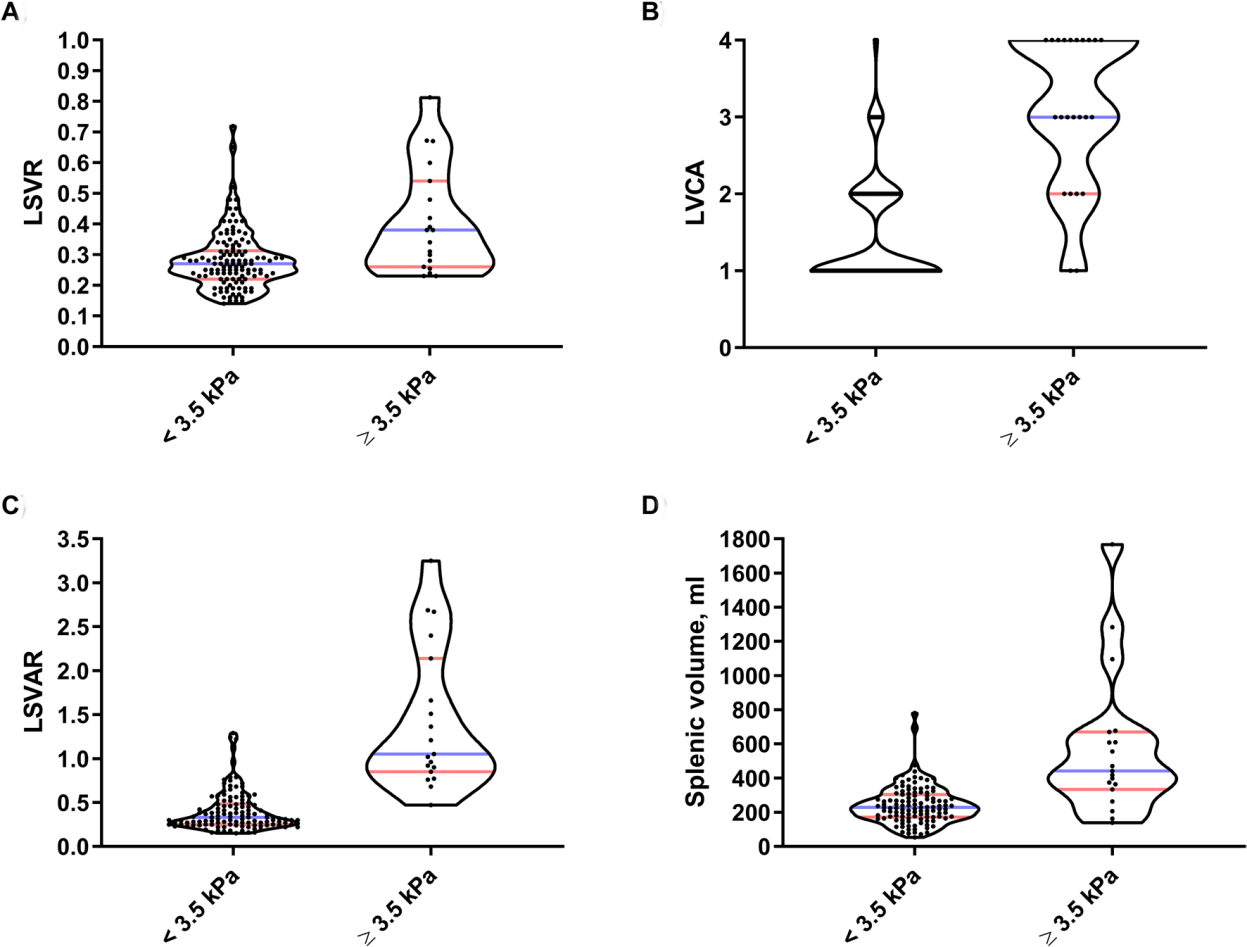
Violin plots comparing patients with and without elevated liver stiffness. Distribution of the results for the LSVR (**a**), splenic volume (**b**), LSVAR (**c**), and LVCA (**d**) are shown for patients with a liver stiffness of < 3.5 and ≥ 3.5 kPa. *ROC* receiver operating characteristic, *AUC* area under the (ROC) curve, *LVCA* liver vein to cava attenuation, *LSVR* liver segmental volume ratio, *LSVAR* liver segmental volume and attenuation ratio

**Fig. 5:**
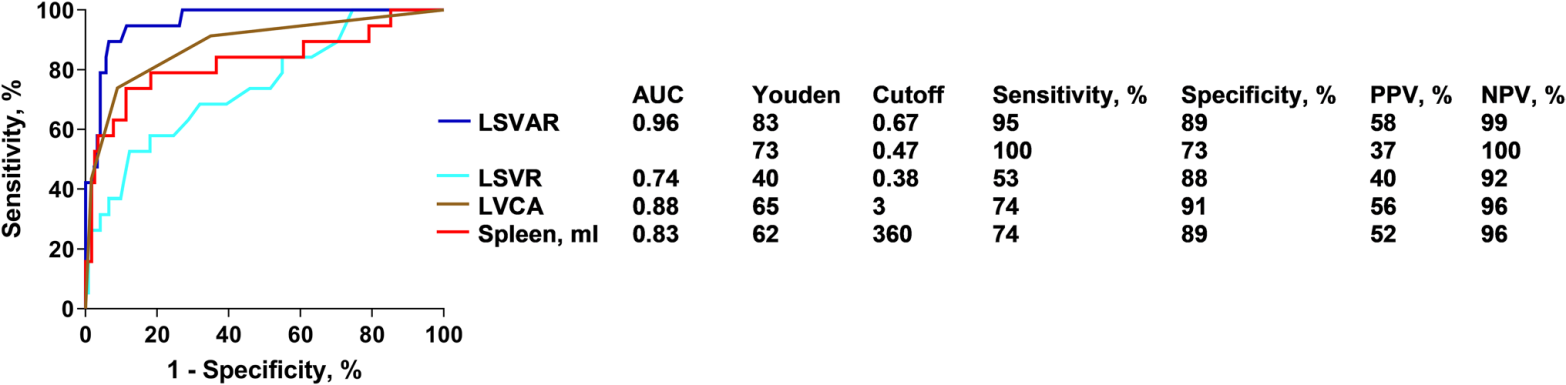
ROC curve analysis. ROC curves of the quantitative CT parameters for distinguishing between patients with and without elevated liver stiffness (shear modulus < 3.5 kPa vs. ≥ 3.5 kPa) are shown. AUC values as well as optimal cutoff values based on Youden’s index with sensitivity and specificity are indicated. An LSVAR cutoff of 0.42 or higher provides a sensitivity of 92% and a specificity of 70%. *ROC* receiver operating characteristic, *AUC* area under the (ROC) curve, *LVCA* liver vein to cava attenuation, *LSVR* liver segmental volume ratio, *LSVAR* liver segmental volume and attenuation ratio

The LSVR, LVCA and LSVAR were all significantly elevated in patients with liver stiffness ≥ 3.5 kPa, as shown in Table 2 and illustrated in violin plots in Fig. 4. In the ROC curve analysis, the LSVR (AUC = 0.74) showed slightly worse performance than splenic volume (AUC = 0.83), but clearly better performance when combined with the LVCA (AUC = 0.88) to obtain the LSVAR (AUC = 0.96) (Fig. 5). The optimal cutoff value for the LSVR was 0.34, and this value predicted clinically significant liver fibrosis with a sensitivity of 53% and a specificity of 88% (10 true-positives, 107 true-negatives, 15 false-positives and 9 false-negatives). The optimal cutoff value for the LSVAR was 0.67, and this value predicted clinically significant liver fibrosis with a sensitivity of 95% and a specificity of 89% (18 true-positive, 109 true-negatives, 13 false-positives and 1 false-negative). Further details are shown in Table 3.

**Table 2:** Descriptive statistics for fibrosis scores

Median [IQR]	Normal to slightly increased liver stiffness (shear modulus ≤ 3.5 kPa)	*n*	Clinically significant increased liver stiffness (shear modulus ≥ 3.5 kPa)	*n*	*p*–value
LVCA	1.00 [1.00–2.00]	122	4.00 [3.00–4.00]	19	< 0.001**
LSVR	0.27 [0.22–0.31]	122	0.38 [0.26–0.54]	19	< 0.001**
LSVAR	0.33 [0.25–0.48]	122	1.05 [0.85–2.14]	19	< 0.001**
I–III, ml	333 [261–396]	122	449 [362–650]	19	0.002*
IV–VIII, ml	1237 [1088–1427]	122	1160 [1008–1579]	19	0.864
Total liver volume, ml	1588 [1371–1844]	122	1743 [1427–2078]	19	0.121
Splenic volume, ml	229 [172–303]	115	442 [333–669]	19	< 0.001

Values are reported as the median and interquartile range. *P* values were calculated using the Mann–Whitney U test. Comparisons between the two patient groups are indicated with * if *p* < 0.05 and ** if *p* < 0.001

*IQR* inter quartile range, *LVCA* liver vein to cava attenuation, *LSVR* liver segmental volume ratio, *LSVAR* liver segmental volume and attenuation ratio

**Table 3:** Diagnostic accuracy for fibrosis scores

Score	LSVR	LSVAR cutoff 0.67	LSVAR cutoff 0.47	LVCA	Splenic volume
True-positive	10	18	19	14	14
True-negative	107	109	89	111	109
False-positive	15	13	33	11	13
False-negative	9	1	0	5	5

The performance to detect significant liver fibrosis for each score is presented in terms of true-positive, true-negative, false-positive and false-negative results. Two different cutoff values for LSVAR are shown. The higher cutoff value is optimized according to Youden’s index in the ROC curve analysis, and the lower cutoff value is optimized for a minimal number of false-negatives

*LSVR* liver segmental volume ratio, *LSVAR* liver segmental volume and attenuation ratio, *LVCA* liver vein to cava attenuation, *ROC* receiver operating characteristic

### Comparison of semiautomated and manual segmentation

The LVCA score was determined in a few seconds in most cases with visual assessment and was still determined in less than one minute in cases with measurements of Hounsfield units. The LSVR and LSVAR were calculated in 10 min using the semiautomated method. In the second analysis with manual segmentation of the liver segments I–III and IV–VIII, the reconstruction time was much longer and took up to 30 min for the LSVR and LSVAR. However, the results remained unchanged with an AUC = 0.74 for the LSVR and an AUC = 0.96 for the LSVAR to predict significant liver fibrosis.

### Interobserver reliability

Interobserver reliability within our group was excellent for the LVCA (ICC = 0.92), LSVR (ICC = 0.96) and LSVAR (ICC = 0.97).

## Discussion

This study shows that the LSVAR improves the detection of significant liver fibrosis on portal venous CT scans by combining the LVCA with the LSVR. The LSVAR may be easily calculated based on standard liver segmental volumetry, and the LVCA can be obtained by simply comparing the liver vein density to the density of the inferior vena cava on any portal venous abdominal CT scan.

A high LVCA reflects delayed enhancement of the liver veins compared to the systemic enhancement of the inferior vena cava. Delayed microperfusion of the liver in chronic liver disease might therefore be a possible explanation for the high LVCA score in patients with significant liver fibrosis. Reduced liver perfusion in chronic liver disease has already been investigated by other groups [19, 20]. As the portal venous perfusion decreases, the arterial perfusion fraction increases, and total liver perfusion is delayed in patients with higher degrees of fibrosis [21]. Zissen et al. reported a significant decrease in the liver-to-aorta ratio in cirrhotic patients compared with patients without CLD [22], while Koiwahara et al. demonstrated a decreasing parenchymal density (ΔHU precontrast to portal venous phase) in patients with higher degrees of chronic liver damage [23].

The LSVR is calculated based on liver segmental volumetry and was initially described by Furusato Hunt et al. [12] based on known liver remodeling in chronic liver disease including volume loss in Couinaud the segments IV-VIII and hypertrophy of segments I-III. The LSVR was significantly increased in patients with liver cirrhosis (LSVR = 0.55) compared with healthy controls (LSVR = 0.27). Compared to Furosato Hunt et al., we found exactly the same median LSVR value in patients with liver stiffness < 3.5 kPa, while the LSVR in patients with increased liver stiffness (≥ 3.5 kPa) was slightly lower in our study. This may be explained by the fact that a stiffness of 3.5 kPa on MR elastography corresponds to a histologic fibrosis score of F2 or higher, while Furusato Hunt et al. included only patients with cirrhosis (F4). Another liver volumetry study showed increased LSVR values in patients with liver fibrosis including those in intermediate stages and revealed values of 0.25 for stage F1, 0.33 for stage F2, 0.39 for stage F3 and 0.56 for stage F4, which reflects the range of our results [11]. In another study by the same group, the diagnostic accuracy was slightly higher, discriminating liver fibrosis stage F0-1 from stage F2-4 with an AUC of 0.85 (the AUC was 0.74 in our study). This might be explained by the larger number of patients with advanced fibrosis [24] in the study by Pickhardt et al., while the present study included a large number of patients without elevated liver stiffness. Pickhardt et al. achieved an even higher diagnostic accuracy for significant fibrosis (≥ F2) with an AUC of 0.91 by combining the LSVR with six other CT features (LSN, periportal space distance, ratio of the left to right portal vein diameters, total liver volume, splenic volume and texture analysis). By finally adding the APRI and FIB-4 score, the AUC could be increased to 0.93, which is similar to the combination of LVCA and LSVR in our study (AUC = 0.96).

Manual segmentation of liver segments I-III and IV-VIII with the region growing tool did not improve the predictive value of the LSVR and LSVAR to detect significant liver fibrosis compared with the semiautomated segmentation. We therefore conclude that the faster, semiautomated method is sufficient to determine the LSVR and LSVAR. Therefore, the LSVR may be calculated in approximately 10 min, and the additional LVCA is determined easily in less than 10 s to calculate the LSVAR. New AI-based algorithms may allow even faster quantification of liver segmental volumes in the near future.

In our study, splenic volume also allowed for the prediction of significantly elevated liver stiffness but had inferior performance to the LVSAR. Measurements of splenic diameter have already been investigated in the 1980s [25]. More recently, splenic volume was shown to detect significant liver fibrosis (≥ F2) with AUC values of 0.85 and 0.76 found by Pickhardt et al. [11, 24], while Lotan et al. found that the splenic volume discriminated between patients with advanced (F2) and severe (F3-F4) fibrosis with an AUC of 0.80 [26], which is within the same range of our presented results (AUC = 0.83). Notably, several nonhepatic factors can cause splenomegaly, and these factors should be taken into account, especially in the setting of CT exams performed for nonhepatic reasons.

The relatively small number of patients with significant fibrosis constitutes a limitation of this paper. The smaller group of 15% with significantly increased liver stiffness and the larger group of 85% without significantly increased liver stiffness represent the spectrum of our center. Therefore, there may be a spectrum bias if the results are compared to those from other centers. In this proof-of-concept study, the LSVAR was a useful predictor for significant liver fibrosis on routine abdominal CT scans in a realistic scenario in a radiology department. The ability of the LVCA and LSVAR to differentiate between degrees of liver fibrosis should now be validated in a larger study including patients with biopsy-proven fibrosis. Another limitation is possible confounding factors unrelated to liver fibrosis that may influence liver stiffness on MR elastography, and the LVCA, such as inflammation in alcoholic and nonalcoholic steatohepatitis [27]. However, MR elastography has been proven to show an excellent correlation with histological fibrosis grade [28] and liver biopsy also has its limitations with important interrater variability and the risk of sampling error [29]. Using a cutoff for liver stiffness of 3.5 kPa or higher, observed changes are so distinct that elevated liver stiffness cannot be explained by inflammation alone. The LVCA might be influenced in cases of contrast reflux in patients with right ventricular dysfunction or tricuspid regurgitation. We therefore excluded the patient with cardiac dysfunction, and there were no further patients with known heart failure in our study population. Contrast reflux into the IVC is mostly seen during the arterial phase; it is therefore important to calculate the LVCA and LSVAR only during correctly timed portal venous phases of the liver. The LVCA was determined visually in most patients by comparing the density of the liver vein with that of the vena cava. Only in unclear cases the densities of the liver veins and the vena cava were measured, while a difference of 20 HU or more was defined as significantly different density between the liver veins and the vena cava. This visual interpretation makes determination of the LVCA very fast. However, since it is qualitative, it may be subject to reader interpretation. Nevertheless, with an ICC of 0.92, the interobserver reliability for the LVCA was excellent. Finally, the LVCA might be influenced by other factors such as a smaller diameter of liver veins in cirrhosis [13] or cardiac output and bolus timing. Such contrast timing differences might be reduced because the LVCA is a relative ratio and not an absolute measurement of Hounsfield units. In any case, the LVCA and LSVAR seem to be robust and reliable imaging biomarkers, which are fast and easy to calculate. While currently dedicated software solutions are needed for liver volumetry, new AI algorithms will overcome this limitation.

In conclusion, the LSVAR improves the detection of significant liver fibrosis on portal venous CT scans compared to the LSVR. This ratio is easy to calculate based on liver segmental volumetry and by comparing the enhancement of the liver veins and the inferior vena cava. The use of the LSVAR allows for the accurate screening of portal venous CT scans to detect possible liver fibrosis with excellent interobserver reliability.

## Data Availability

Anonymized data files may be sent on request.

## Abbreviations

AUC: Area under the curve
CLD: Chronic liver disease
CRL-R: Caudate–right lobe ratio
HCC: Hepatocellular carcinoma
IVC: Inferior vena cava
LIMA-FS: Liver imaging morphology and attenuation fibrosis score
LSVR: Liver segmental volume ratio
LSVAR: Liver segmental volume and attenuation ratio
LVCA: Liver vein to cava attenuation
MDCT: Multidetector computed tomography
MR: Magnetic resonance
ROC: Receiver operating characteristics
